# Supplementary material for: A Light/Pressure Bifunctional Electronic Skin Based on a Bilayer Structure of PEDOT:PSS-Coated Cellulose Paper/CsPbBr3 QDs Film
Source: Polymers (Basel). 2023 Apr 29;15(9):2136. doi: 10.3390/polym15092136 (PMC10181253; doi:10.3390/polym15092136)
Supplement: Supplementary file 1 [file polymers-15-02136-s001.zip › polymers-2359616-supplementary.pdf]

Article

A Light/Pressure Bifunctional Electronic Skin Based on a Bilayer Structure of PEDOT:PSS-Coated Cellulose Paper/$CsPbBr_3$ QDs Film

Wenhao Li [1], Jingyu Jia [1], Xiaochen Sun [1], Sue Hao [1,*] and Tengling Ye [1,2,*]

[1] Department of Applied Chemistry, School of Chemistry and Chemical Engineering, Harbin Institute of Technology, Harbin 150001, China; liwenhao0824@163.com (W.L.); jjy271828@163.com (J.J.); 15684172397@163.com (X.S.)

[2] CAS Key Laboratory of Renewable Energy, Guangzhou Institute of Energy Conversion, Guangzhou 510640, China

* Correspondence: haosue@hit.edu.cn (S.H.); ytl@hit.edu.cn (T.Y.)

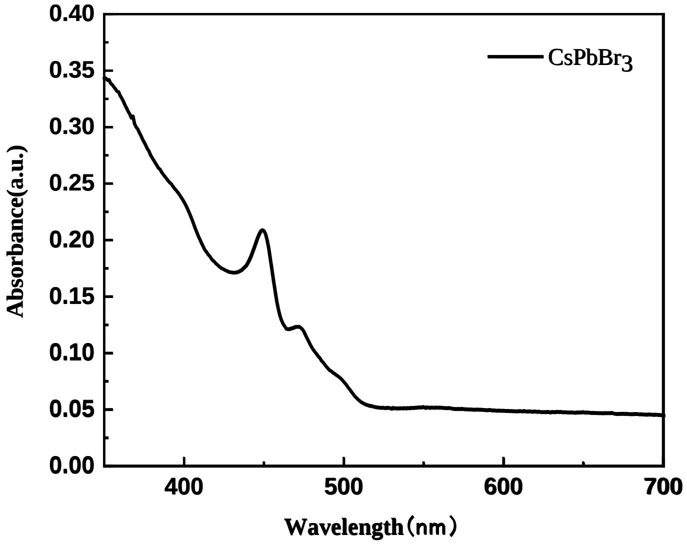

Figure S1. The absorption spectrum of $CsPbBr_3$ QDs in n-hexane solution.

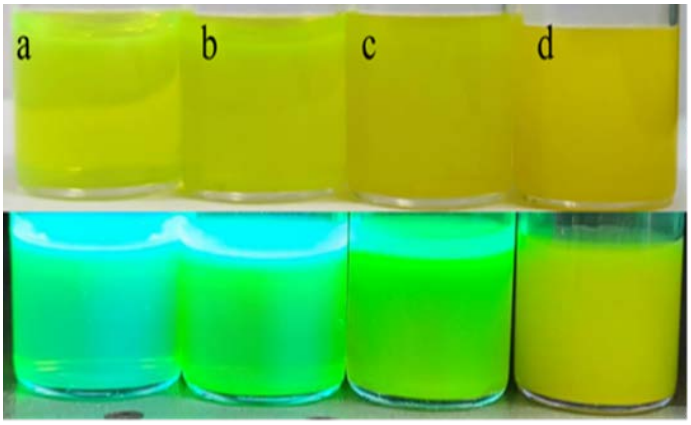

Figure S2. Solutions of $CsPbBr_3$ QDs washed by different solvents under fluorescent lamps (upper photos) and under 365 nm UV lamps (lower photos): (**a**) without washing; (**b**) washed by ethyl acetate; (**c**) washed by isopropanol; (**d**) washed by acetone.

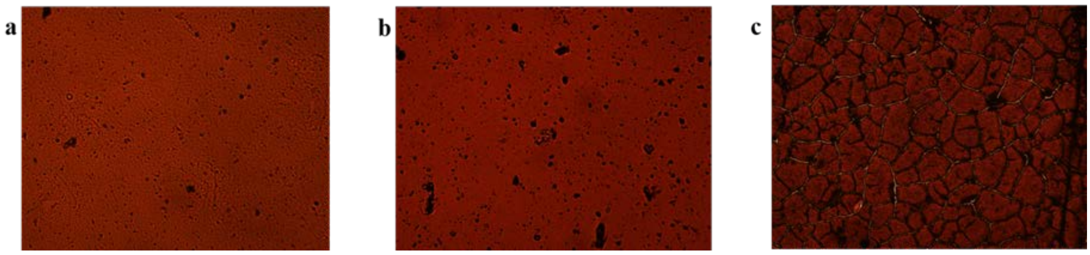

Figure S3. Microscopic morphologies of photodetectors washed by different solvents: (**a**) ethyl acetate, (**b**) isopropyl alcohol, (**c**) acetone (160x magnification).

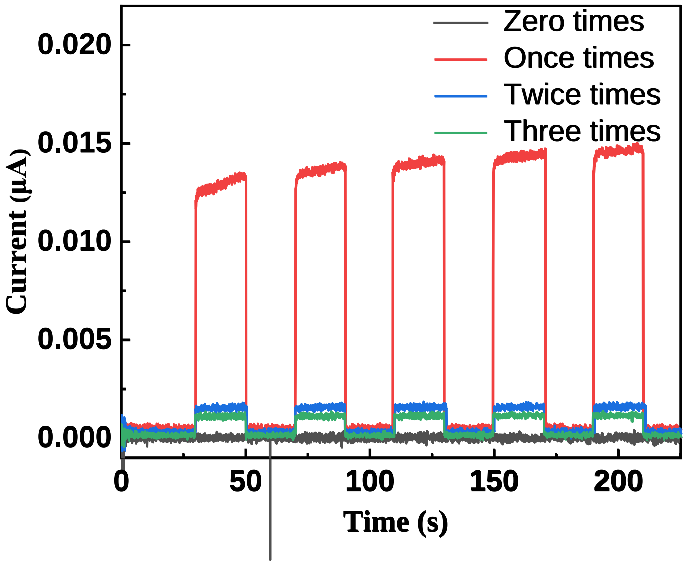

Figure S4. I-t curves of photodetectors under different washing times (405 nm, 10v) .

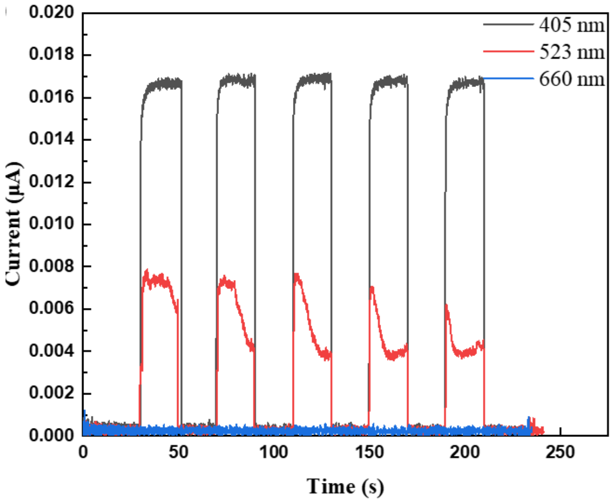

Figure S5. I-t curves of photodetectors under different wavelengths (40 mW/cm^2,10v).

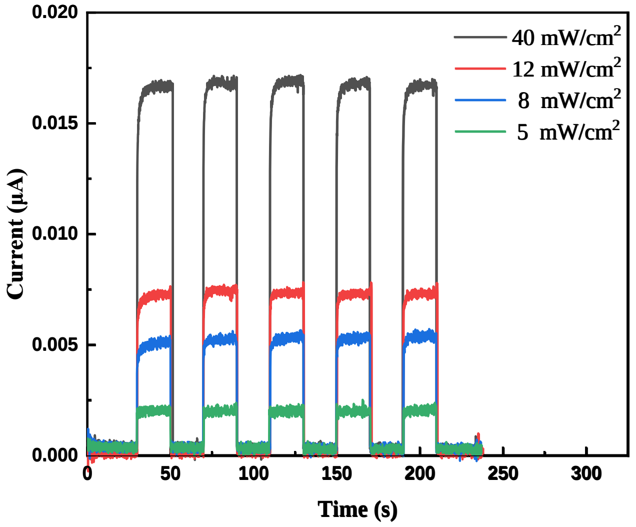

Figure S6. I-t curves of photodetectors under different optical power density (405 nm, 40 mW/cm²).

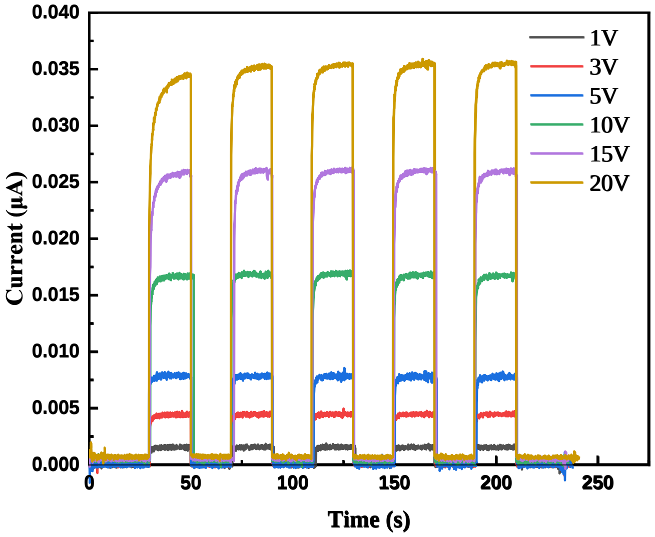

Figure S7. I-t curves of photodetectors at different voltages (405 nm, 10v).

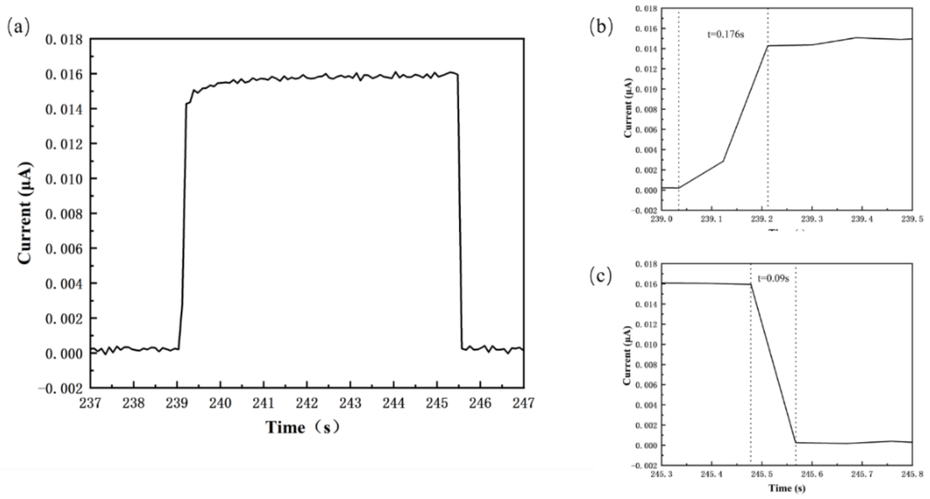

Figure S8. One cycle of photodetector and its response time（b is light on and c is light off）.

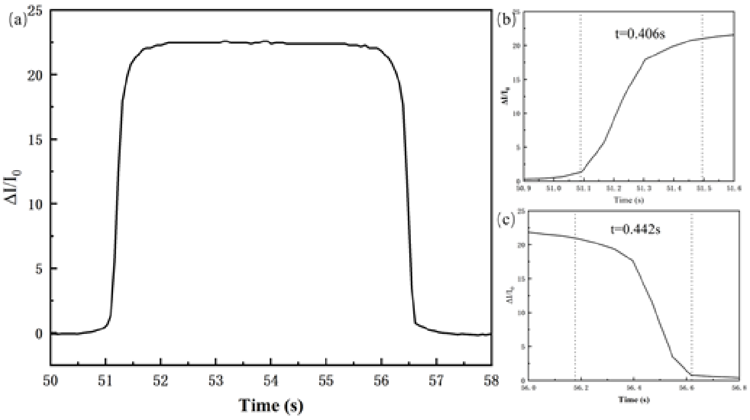

Figure S9. One cycle of pressure sensor and its response time（b is pressure on and c is pressure off）.

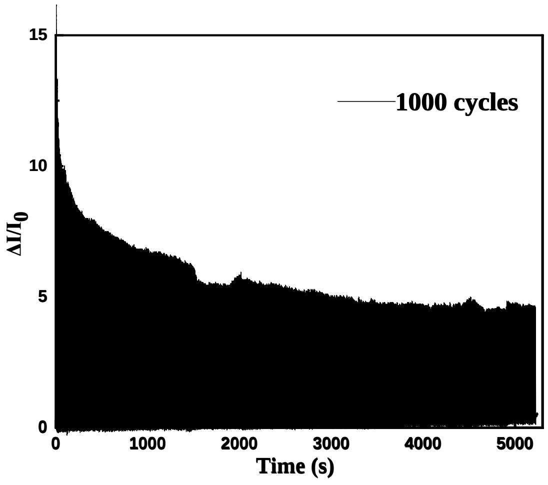

Figure S10. $\Delta I/I_0$ vs t curves of the pressure sensor (1000 cycles); all applied bias is 1 v.
